# “Clone-specific” antibody-drug conjugates: an innovative strategy in the treatment of T-cell cancers

**DOI:** 10.1038/s41392-024-01945-7

**Published:** 2024-09-04

**Authors:** Dennis Jungherz, Philipp Lückemeier, Marco Herling

**Affiliations:** Department of Hematology, Cellular Therapy, Hemostaseology and Infectious Diseases, University of Leipzig Medical Center, Leipzig, Germany

**Keywords:** Haematological cancer, Drug development, Drug delivery

In a recent study published in *Nature*, Nichakawade et al. provided proof-of-principle data on innovative strategies to target and eliminate cancerous T-cells while sparing healthy T-lymphocytes, addressing the urgent clinical need posed by the mostly aggressive and treatment-refractory T-cell lymphomas.^1^ This research represents a significant milestone in overcoming major obstacles in immunotherapies for these cancers.

Choosing the right target: Approaches like antibody-drug conjugates (ADC) or CAR-T-cells have revolutionized the treatment of B-cell lymphomas. However, T-cell malignancies have drastically less benefited from these advancements. Reasons for this are their rarity and marked heterogeneity. Hence, identifications of highly discriminatory and targetable antigens have been challenging in these hematologic neoplasms. A notable exception is the ADC Brentuximab-vedotin (BV) that is directed against the surface protein CD30, which is particularly abundant in anaplastic large cell lymphoma (ALCL) and only rarely expressed on normal T-cells. The success of BV illustrates the potential of precise targeting of quasi-selective T-cell lymphoma antigens. By delivering its microtubule disrupting payload MMAE directly to CD30^+^ cancer cells, BV, meanwhile, an established standard in first-line or second-line settings in ALCL, has significantly improved treatment efficacy and patient survival while minimizing toxicity, both in combination with chemotherapy or as a single agent.^2^

Another challenge in devising T-cell targeted therapies without good discrimination between malignant vs normal T-cells is the therapy-related T-cell hypoplasia that results in severe immunosuppression, thought to be of more life-threatening potential than B-cell depletion.

Mode of execution: When using T-cells as effector cells (i.e., a CAR-T-cell approach) against T-cell tumors there are additional hurdles to overcome. First, in-vitro and in-patient expansion can be severely impaired by self-directed killing among CAR-T-cells that express that therapeutic antigen themselves - the so-called fratricide. A second shortcoming of using autologous T-cell harvests as the source for therapeutic (CAR) T-cells is product contamination by malignant T-cells with all the implications around their immunologically hypo-functional state or the transduction of another growth-promoter (CAR) potentially propelling clonal outgrowth. Moreover, prior T-cell depleting (chemo)therapies limit the yield of competent CAR-T-cells. Finally, the clinical scenarios of refractory T-cell malignancies often dictate the immediate application of effective salvage strategies followed by the swift application of the more definitive cell-therapeutic approach. Here, off-the-shelf solutions would overcome the limitations by the time-intensive process of CAR-T-cell production.

**Fig. 1:**
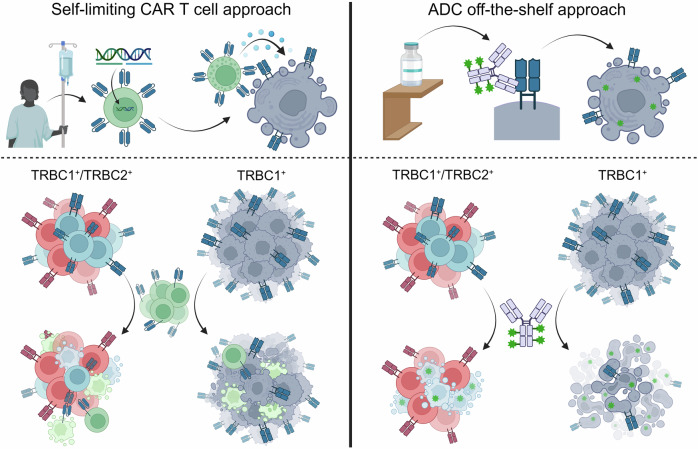
Approaches to target TRBC1. Left: Patient-derived autologous T-cells are genetically modified to TRBC1-targeting CAR-T-cells (green) and retransfused into the patient. Besides, eradicating both normal TRBC1^+^ (light blue) as well as malignant TRBC1^+^ T-cells (dark blue), anti-TRBC1 CAR-T-cells are killed by normal TRBC1^+^ T-cells as well (self-limiting). Right: Monoclonal anti-TRBC1 antibodies are conjugated (ADC) to cytotoxic drugs (green asterisk). Similarly, anti-TRBC1 ADC targets normal TRBC1^+^ (light blue) and malignant TRBC1^+^ T-cells (dark blue). In both scenarios, untargeted normal TRBC2^+^ T-cells (red) ensure the patients’ immune competence. Created with BioRender.com

The authors around Nichakawade et al. devised a strategy that has the potential to overcome many of these limitations. They built on seminal work by Maciocia et al. who identified the T-cell receptor β-chain constant region 1 (TRBC1) as a promising target for treating T-cell cancers.^3^ Due to its clonal restriction on cancerous T-cells (either only TRBC1 or only TRBC2) while healthy T-cell repertoires being comprised of a 1:2-mixture of TRBC1^+^:TRBC2^+^ T-cells, a TRBC-targeted immunotherapy would eradicate the monoclonal malignant T-cell population while preserving sufficient normal T-cells (Fig. 1). Complementing the highly specific and promising anti-TRBC1 antibody, Ferrari et al. also established a TRBC2-specific antibody with promising therapeutic potential.^4^ This introduces the possibility of targeting the majority of mature T-cell lymphomas by options of a TRBC1 or -2-based approach conferring quasi-anti-clonal selectivity with minimized damage to normal T-cell repertoires. Of note, clinical trials utilizing anti-TRBC1 CAR-T-cells encountered relevant challenges; e.g., they reported an unexplained loss of CAR-T-cells in patients.^5^

Nichakawade et al. add here by identifying a rejection phenomenon in which normal TRBC1^+^ T-cells kill anti-TRBC1 CAR-T-cells, as a factor that could limit the efficacy of anti-TRBC1 CAR-T-cells. This finding emphasizes the complexities involved in developing sustainable CAR-T-cell strategies for T-cell cancers. As an attractive alternative, this study carries on with the idea of ADCs to target TRBC1 and -2, as already technically explored by Ferrari et al.^4^ ADCs combine the specificity of monoclonal antibodies with the cytotoxic potency of their payload(s). The researchers of Nichakawade et al. conjugated monoclonal anti-TRBC1 antibodies to the cytotoxic MMAE (‘vedotin’) and the cytotoxic drug SG3199 (a cytotoxic DNA minor groove inter-strand crosslinking pyrrolobenzodiazepine (PBD) dimer), resulting in an anti-TRBC1-SG3249 ADC. The study conducted extensive preclinical testing to evaluate the efficacy and specificity of the created anti-TRBC1-SG3249 ADC. In-vitro experiments demonstrated potent cytotoxicity against TRBC1^+^ cancer cells, with minimal impact on normal T-cells expressing TRBC2. These findings were further validated in mouse models of human T-cell cancers, where the anti-TRBC1-SG3249 ADC exhibited significant activity and high potential to eradicate disseminated T-cell proliferation.

Overall, Nichakawade et al. elegantly demonstrate that TRBC1-ADCs can efficiently bypass the bespoke manufacturing as well as rejection challenges by anti-TRBC1 CAR-T-cell products, which may lead to faster treatment initiations and improved outcomes over those by autologous T-effector cell approaches in patients with T-cell cancers. TRBC1-ADCs offer a novel approach to spare large proportions of the healthy T-cell repertoire. Considering the expensive, time-consuming, and complex production of autologous CAR-T-cells, ‘off-the-shelf’ ADCs allow a faster administration hence lowering the chance of disease progression during error-prone CAR production processes. Further research will be needed to validate these findings and to translate them into clinical applications.

In this context, a set of issues remains to be addressed: There are T-cell malignancies that lost their surface T-cell receptor rendering the described targeted approaches ineffective. Moreover, what would be related escape modes of TRBC1- or -2 expressing tumors while under treatment with an anti-TRBC1/2 ADC? Furthermore, there is growing evidence suggesting TRBC1/2 oligoclonality among some T-cell lymphomas, which would further limit the effectiveness of this design. Also, their heterogeneity and wide-spread resistance to conventional (chemo)therapies (hence payloads?) would implicate that sensitivities to the TRBC1/-2 approach could vary greatly across T-cell-lymphoma subsets. This also pertains to their heterogeneous tropism in association to differential tissue penetration of an ADC at specific sites of presentation (e.g., gut, skin, etc.) as compared to CAR-T-cells. Lastly, trial designs (e.g., scheduling) will have to consider that ADCs are short-lived and need to be re-applied, unlike CAR-T-cells as ‘living drugs’. We are convinced that we will eventually see a TRBC1/-2 ADC approach in several settings, e.g., as part of combination strategies, as a bridging in low-burden disease before an allogeneic stem cell transplantation, or as a maintenance concept.
